# *Aspergillus niger* Ochratoxinase Is a Highly Specific, Metal-Dependent Amidohydrolase Suitable for OTA Biodetoxification in Food and Feed

**DOI:** 10.1021/acs.jafc.4c02944

**Published:** 2024-08-07

**Authors:** Ana Sánchez-Arroyo, Laura Plaza-Vinuesa, Blanca de las Rivas, José Miguel Mancheño, Rosario Muñoz

**Affiliations:** † Bacterial Biotechnology, Institute of Food Science, Technology and Nutrition (ICTAN), CSIC, José Antonio Novais 6, 28040 Madrid, Spain; ‡ Department of Crystallography and Structural Biology, Institute of Physical Chemistry Blas Cabrera (IQF), CSIC, Serrano 119, 28006 Madrid, Spain

**Keywords:** mycotoxin, additive, ochratoxin, amidohydrolase, carboxypeptidase

## Abstract

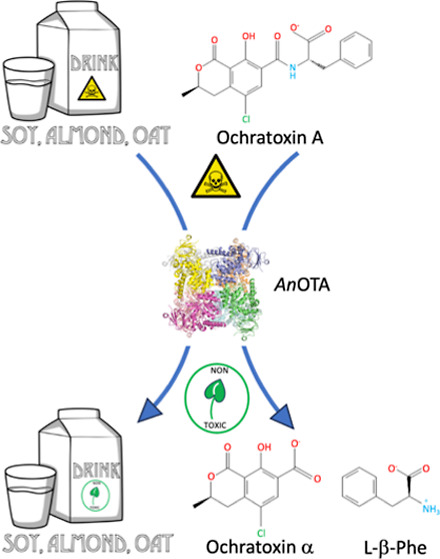

Microbial enzymes can be used as processing aids or additives in food and feed industries. Enzymatic detoxification of ochratoxin A (OTA) is a promising method to reduce OTA content. Here, we characterize the full-length enzyme ochratoxinase (*An*OTA), an amidohydrolase from *Aspergillus niger*. *An*OTA hydrolyzes OTA and ochratoxin B (OTB) mycotoxins efficiently and also other substrates containing phenylalanine, alanine, or leucine residues at their C-terminal position, revealing a narrow specificity profile. *An*OTA lacks endopeptidase or aminoacylase activities. The structural basis of the molecular recognition by *An*OTA of OTA, OTB, and a wide array of model substrates has been investigated by molecular docking simulation. *An*OTA shows maximal hydrolytic activity at neutral pH and high temperature (65 °C) and retained high activity after prolonged incubation at 45 °C. The reduction of OTA levels in food products by *An*OTA has been investigated using several commercial plant-based beverages. The results showed complete degradation of OTA with no detectable modification of beverage proteins. Therefore, the addition of *An*OTA seems to be a useful procedure to eliminate OTA in plant-based beverages. Moreover, computational predictions of in vivo characteristics indicated that *An*OTA is neither an allergenic nor antigenic protein. All characteristics found for *An*OTA supported the suitability of its use for OTA detoxification in food and feed.

## Introduction

Mycotoxins are toxic chemical compounds produced by molds that cause huge economic losses to global agriculture. Ochratoxin A (OTA) is an important mycotoxin mostly found in dried fruits, coffee and cocoa beans, cereals, and spice.^1^ To control OTA contamination, several countries have established regulations for OTA levels in a variety of food products.^2^ Strategies for detoxifying OTA generally include physical, chemical, and biological methods, including enzymatic treatment.^3^ Enzymes are included in EC regulation 386/2009 which establishes a new functional group of feed additives for the reduction of mycotoxin contamination, being substances that can suppress or reduce the absorption, promote the excretion of mycotoxins, or modify their mode of action.^4^ Enzymatic transformation of mycotoxins presents the advantages of high efficiency, strong specificity, and lack of damage to nutrients.^5^

Microbial enzymes are known to play a crucial role in numerous industries and applications. The demand for industrial enzymes is increasing due to the growing need for sustainable solutions. Food enzymes are mainly used to improve food taste, texture, digestibility, and nutritional value, being considered as processing aids. In general, processing aids are used during the food manufacturing process and do not have a technological function demand in the final food. Food enzymes are often not sold as pure enzymes but as enzyme preparations containing not only the desired enzyme but also various added substances. All these components are expected to be safe according to good manufacturing practice guidelines.^6^

In this regard, *Aspergillus* is a ubiquitous fungus found in nature. Species from this genus are widespread, being frequently isolated from soil, plant debris, and indoor air environments. Due to their prevalence in the natural environment, their ease of cultivation on laboratory media, and the economic importance of several of its species, this organism is among the most successful groups of molds involved in many industrial processes.^7^

*Aspergillus niger* is generally regarded as a safe organism and a nonpathogenic fungus widely distributed in nature.^8^*A. niger* is one of the most important microorganisms used in biotechnology, having been used for many decades to produce food enzymes. Since the 1960s, *A. niger* has become a source of a variety of enzymes that are well established as technical aids in fruit processing, baking, and in the starch and food industries.^7^

An *A. niger* enzyme, defined as amidohydrolase 2 (here, *An*OTA), has been described to transform OTA through the hydrolysis of the amide bond to generate OTα and l-β-phenylalanine (Figure S1), virtually nontoxic compounds.^9^ When the enzyme was homologously produced in *A. niger*, the recombinant amidase was N-terminally truncated, lacking the first 42 amino acid residues. This truncated protein was partially characterized and its three-dimensional structure was resolved.^9^ This structure reveals that *An*OTA is a homo-octamer with D4 dihedral symmetry, in which the subunits fold into a two-domain structure characteristic of metal-dependent amidohydrolases.^9^

Since knowledge of the substrate specificity profile is of utmost relevance when an enzyme is to be used in detoxification processes involving complex, protein-rich environments, such as food, feed, and gut, here, we aim to analyze in detail the specificity profile of full-length *An*OTA, extending the analyses carried out previously demonstrating a preference for phenylalanine at the C-terminal end of the scissile amide bond.^9^ Therefore, in this work, a biochemical characterization including the substrate specificity of the full-length *An*OTA, heterologously produced in *Escherichia coli*, was determined by using synthetic substrates. We have also analyzed the structural bases of this substrate specificity by molecular docking simulations. Moreover, the use of *An*OTA for OTA detoxification in plant-based beverages was assayed. Finally, the prediction of some in vivo *An*OTA characteristics was also included in the study. All these results contribute to elucidating the suitability of *An*OTA for its use as food and feed additives.

## Materials and Methods

### Strains and Growth Conditions

The *A. niger* CBS 513.88 strain used in this study was purchased from the Westerdijk Fungal Biodiversity Institute (The Netherlands) and was routinely grown in the media and conditions recommended.

*E. coli* DH10B was used for DNA manipulations. *E. coli* BL21(DE3) was used for protein expression in the pURI3-Cter vector.^10^*E. coli* strains were cultured in Luria–Bertani (LB) medium at 37 °C and shaken. When required, ampicillin was added to the medium at 100 μg/mL.

### Production and Purification of Ochratoxinase from *A. niger* CBS 513.88 (*An*OTA)

The full-length gene encoding *An*OTA (amidase 2) from *A. niger* CBS 513.88 (AM270317.1, contig An14c0100, 6231–7673 positions) was amplified by PCR using oligonucleotides Fw (forward) (5′-*AACTTTAAGAAGGAGATATACATatg*gtccgccgaattgct tcagctac) and Rv (reverse) (5′-*GCTATTAATGATGATGATGATGATG*cagaaaaggattacgtgcat cttc) (the nucleotides pairing the sequence of the expression vector are designated in italics, and the nucleotides pairing the sequence of the gene are designated in lowercase letters). The 1.4-kb amplified PCR product was purified and inserted into the pURI3-Cter vector by a restriction enzyme- and ligation-free cloning method.^10^ This vector delivers recombinant proteins displaying a six-histidine affinity tag in their C-terminal end. *E. coli* DH10B cells were transformed and the recombinant plasmids were purified. Next, they were transformed into *E. coli* BL21(DE3) cells for protein expression. Since the obtained protein yield in the expression assays was low, a pMA plasmid derivative containing the *A. niger* gene with the codon usage adapted to the codon bias of *E. coli* was synthesized by Invitrogen (Thermo Fisher Scientific). The *An*OTA encoding gene with the optimized *E. coli* codon usage was amplified by oligonucleotides Fw and Rv from the pMA plasmid. The PCR fragment was then inserted into the pURI3-Cter vector and transformed into *E. coli* DH10B cells for cloning and, later, into *E. coli* BL21 (DE3) for protein expression.

*E. coli* BL21(DE3) cells harboring the recombinant vector pURI3-Cter-*An*OTA were grown at 22 °C in the LB medium containing 1 M D-sorbitol, 25 mM glycine betaine, and 0.25 mM isopropyl-β-d-thiogalactopyranoside to avoid inclusion body formation.^11^ The cells were disrupted by French press lysis; afterward, the insoluble fraction of the lysate was separated by centrifugation at 47,000*g* for 30 min at 4 °C. The amidase *An*OTA was purified by batch affinity chromatography using TALON Superflow resin (Clontech) and eluted using 50 mM MOPS, pH 7.0, supplemented with 150 mM NaCl and 150 mM imidazole. The eluted His-tagged *An*OTA was dialyzed overnight at 4 °C against 50 mM MOPS buffer, pH 7.0, containing 150 mM NaCl. Enzyme purity was verified by 12.5% SDS-PAGE in Tris-glycine buffer.

### Effect of Temperature and pH on *An*OTA Amidohydrolase Activity

The biochemical characterization of the full-length *An*OTA was performed by using the OTA analogue *N*-(4-methoxyphenylazoformyl)-phenylalanine (4MF) (Bachem, Switzerland) as a substrate.^9^ This activity assay consists of the measurement of loss of absorbance at a wavelength of 350 nm, caused by the hydrolysis of 4MF. A 4MF stock solution (55 mM) was prepared in DMSO. To examine the influence of temperature, reactions were performed with 0.1 mM 4MF in 50 mM buffer MOPS (pH 7.0) containing 20 mM NaCl at 5, 20, 30, 37, 40, 45, 55, 65, 75, and 85 °C, using 100 ng of the enzyme. The reactions were incubated for 15 min, after which the absorbance was measured. The influence of pH was investigated by assaying amidase activity in a set of buffers with pH values from 3.0 to 9.0. The buffers (50 mM) utilized were sodium citrate buffer (pH 3.0–6.0) and Bis-Tris propane buffer (pH 6.5–9.0). Reactions containing 100 ng of the enzyme and 0.1 mM 4MF were incubated at 37 °C for 15 min, after which the absorbance was measured. For the measurement of the thermal stability, *An*OTA was incubated in 50 mM buffer MOPS (pH 7.0) containing 20 mM NaCl at 20, 30, 37, 45, 55, and 65 °C for 15 and 30 min and 1, 2, 4, 6, and 20 h. After incubation, the residual activity was measured. Reaction mixtures with no added enzyme were used as negative controls. All reactions were carried out in triplicates.

### Determination of the Influence of Different Metals on *An*OTA Activity

The effects of the presence of different metal ions were analyzed in untreated, native (*An*OTA^–^), and EDTA-treated *An*OTA (*An*OTA^+^). The chelating agent EDTA was used in an attempt to obtain the metal-free enzyme. Purified, full-length *An*OTA was treated with 20 mM EDTA at room temperature for 2 h to remove metal ions. Subsequently, the chelating agent was removed by extensive, overnight dialysis against 50 mM buffer MOPS (pH 7.0) containing 20 mM NaCl at 4 °C with three changes of buffer.^12^ Reconstitution experiments were performed by adding the metal salt (1 mM) to *An*OTA^+^ and preincubating it at room temperature for 15 min, before the activity assay. Then, 4MF was added to a final concentration of 0.1 mM. The reactions were incubated for 15 min at 37 °C, and the absorbance was then measured. The residual amidase activity was measured after the incubation of *An*OTA with each metal. The compounds assayed were MgCl_2_, ZnCl_2_, NiCl_2_, CoCl_2_, and MnCl_2_. In all assays, the amidase activity measured in the absence of any additive was taken as a control and was given a value of 100%. The experiments were done in triplicate.

### Ochratoxin-Detoxification Assays

Enzymatic transformation of OTA and ochratoxin B (OTB) by *An*OTA was confirmed by HPLC. Stock solutions (1 mg/mL) were prepared dissolving OTA and OTB in methanol and stored at −20 °C. Reactions were performed in phosphate buffer 50 mM (pH 7.0), using 5 μg of the enzyme and adding OTA or OTB to a 5 μM final concentration. Reaction mixtures were incubated at 37 °C overnight and stopped by heating for 5 min at 95 °C. Afterward, samples were centrifuged at 14,000*g* for 5 min. The supernatants were filtered through 0.45 μm syringe filters (Millipore, USA) and analyzed by HPLC as previously described.^13^

### Determination of *An*OTA Substrate Specificity

Endopeptidase activity was evaluated by incubating 1 μg of *An*OTA with 15 μM bovine serum albumin (BSA) in 50 mM MOPS buffer containing 20 mM NaCl (pH 7.0) at 37 °C overnight. A reaction mixture with no added *An*OTA was employed as a negative control. BSA degradation was examined by SDS-PAGE analysis of the reaction mixture after incubation. Coomassie brilliant blue R250 stain was used for gel staining.

Hydrolytic activity on 11 *N*-acetyl-l-amino acids was assayed [*N*-acetyl-l-alanine (AcA), *N*-acetyl-l-phenylalanine (AcF), *N*-acetyl-l-leucine (AcL), *N*-acetyl-l-methionine (AcM), *N*-acetyl-l-tyrosine (AcY), *N*-acetyl-l-asparagine (AcN), *N*-acetyl-l-aspartic acid (AcD), *N*-acetyl-l-glutamine (AcQ), *N*-acetyl-l-glutamic acid (AcE), *N*-acetyl-l-proline (AcP), and *N*-acetyl-l-lysine (AcK)]. Hydrolytic activity on 15 *N*-benzyloxycarbonyl (or carbobenzyloxy) amino acid derivatives was assessed. These substrates were the following: carbobenzyloxy-l-alanine (ZA), carbobenzyloxy-l-phenylalanine (ZF), carbobenzyloxy-leucine (ZL), carbobenzyloxy-isoleucine (ZI), carbobenzyloxy-methionine (ZM), carbobenzyloxy-tyrosine (ZY), carbobenzyloxy-serine (ZS), carbobenzyloxy-asparagine (ZN), carbobenzyloxy-glutamine (ZQ), carbobenzyloxy-aspartic acid (ZD), carbobenzyloxy-glutamic acid (ZE), carbobenzyloxy-lysine (ZK), carbobenzyloxy-proline (ZP), carbobenzyloxy-l-alanyl-l-phenylalanine (ZAF), carbobenzyloxy-l-phenylalanyl-l-isoleucine (ZFI), carbobenzyloxy-l-alanyl-l-leucine (ZAL), carbobenzyloxy-β-alanyl-l-alanine (ZAA), and carbobenzyloxy-l-isoleucyl-l-phenylalanine (ZIF). The activity on glycyl-l-phenylalanine (GF), glycyl-l-alanine (GA), *N*-benzoyl-glycyl-phenylalanine (hippuryl-phenylalanine, HF), and *N*-benzoyl-glycyl-arginine (hippuryl-arginine, HR) was also studied. All these substrates were purchased from Sigma-Aldrich (Germany). A stock solution of each substrate (25 mM) was prepared in water or ethanol depending on their solubility. The reactions were performed in MOPS buffer 50 mM (pH 7.0) containing 20 mM NaCl, using 5 μg of the enzyme and adding each substrate to a final concentration of 1 mM. The reactions were incubated for 1 h at 37 °C. The hydrolytic activity was studied by the modified Cd-ninhydrin method as described before.^14^ This method measures free amino acids in a solution using a colorimetric reaction. The formation of the pink compound between the Cd-ninhydrin reagent and the free-amino acids released from the hydrolysis reaction was measured at 507 nm in a UVmini-1240 spectrophotometer (Shimadzu). For substrates containing proline, the yellow compound formed between the Cd-ninhydrin reagent and the free l-proline released was measured at 440 nm.^15^ A calibration curve for each amino acid was built by analyzing samples containing each standard amino acid at concentrations ranging from 0.1 to 1 mM with the Cd-ninhydrin method. Reaction mixtures with no added enzyme were used as negative controls. The assays were conducted in triplicate.

### OTA Detoxification in Plant-Based Beverages by *An*OTA

Three commercial UHT plant-based beverages (almond, oat, and soy) were purchased from a local supermarket (Hacendado, Mercadona, Spain). The composition and nutritional values of the beverages are stated on their labels. Almond beverage contained 3% almond and 0.5% protein; oat beverage contained 8% oat and 0.7% protein, and soy beverage contained 13% soybean and 3.1% protein. All samples contained other secondary ingredients such as stabilizers, thickeners, aromas, salt, and sugars. The protein content of the plant-food beverages was spectrophotometrically determined at 260 nm in a NanoDrop spectrophotometer.

The OTA detoxification assay of the three plant-based beverages was performed as described previously. Reactions were performed in the beverages by adding OTA at 5 μM final concentration and using 5 μg of the *An*OTA enzyme. Reactions were incubated at 20 and 37 °C for 4 h. After incubation, OTA was extracted by using an OchraTest WB (VICAM, USA) immunoaffinity column, following the protocol described for cereal samples. Briefly, 2 mL of the beverage sample was diluted with 8 mL of methanol. The solution was mixed under stirring for 15 min. Next, it was passed through a filter paper. The filtered extract was then diluted with 40 mL PBS (phosphate buffered saline) and mixed well. The extract was filtered through a 1.5 μm glass microfiber filter (VICAM, USA). A 10 mL volume of diluted extract was applied to an OchraTest WB immunoaffinity column at a flow rate of about 1 drop/second. The column was washed with 10 mL of PBS followed by 10 mL of distilled water. OTA or its degradation product, OTα, was eluted with 1.5 mL of methanol and collected in a clean vial. The eluted extract was evaporated overnight at 60 °C and reconstituted with 500 μL of the HPLC mobile phase.

Protein profiles of plant-based beverages were examined by SDS-PAGE analysis of the reaction mixtures after incubation. Coomassie brilliant blue R250 stain was used for gel staining. To determine the free-amino acid composition of the plant-based beverages treated with *An*OTA (15 μg of enzyme, 4 h at 37 °C) and nontreated, in the first place, after the incubation period, samples were freeze-dried using a Beta 2–8 LDplus (Christ, Germany) lyophilizer. Subsequently, free-amino acids were extracted by shaking 0.3 g of the freeze-dried sample with 2 mL of HCl 0.1 M during 1 h. The samples were then centrifuged at 12,000*g* for 10 min at 4 °C and the supernatant was filtered through a 0.45 μm filter (Millipore, USA). The free-amino acid composition was evaluated by the use of a Biochrom 30 amino acid analyzer. The Biochrom 30 uses the classical amino acid analysis methodology based on ion-exchange liquid chromatography and postcolumn continuous reaction with ninhydrin to provide qualitative and quantitative compositional analysis. The amino acid composition analysis was carried out in triplicate at the Protein Chemistry facility of the Center for Biological Research (CIB-CSIC).

### Molecular Docking Simulations

Molecular docking was performed using AutoDock Vina^16^ and UCSF Chimera 1.17^17^ as the interface for the preparation of pdbqt files. The crystal structure of the truncated *An*OTA determined at 2.5 Å resolution (PDB code: 4C5Z) was used as the receptor molecule. Its preparation for docking experiments was as reported before^13^ and involved the removal of waters, addition of polar hydrogens, and merging of charges. The atomic coordinates were retrieved from the PubChem database (https://pubchem.ncbi.nlm.nih.gov/) for the substrates ZF (PubChem CID: 70878), ZI (PubChem CID: 2724772), ZA (PubChem CID: 736104), HF (PubChem CID: 6994977), and HR (PubChem CID: 96815). The rest of the structures, namely, ZAF, ZAL, ZAA, ZIF, and ZFI, were built as described previously.^13^ The structures of the substrates were energy minimized with USCF Chimera (default values) before the docking experiments. A search exhaustiveness of 32 and a maximum energy difference of 3 kcal/mol were used. The number of binding modes was 10. USCF Chimera^17^ and PyMOL^18^ were used for the visualization of the structures, analysis of the interactions, and figure preparation.

### Computational Prediction of In Vivo Protein Characteristics

The allergenicity of *An*OTA was predicted using the web tools AllerTOP v 2.0 (http://www.ddg-pharmfac.net/AllerTOP/index.html)^19^ and AlgPred 2.0 (http://crdd.osdd.net/raghava/algpred/).^20^ The VaxiJen program (http://www.ddg-pharmfac.net/vaxijen/VaxiJen/VaxiJen.html) was applied for the prediction of antigenicity.^21^ NetMHCcons (http://www.cbs.dtu.dk/services/netMHCcons/) and NetMHCIIpan (http://www.cbs.dtu.dk/services/NetMHCIIpan/) were used for the prediction of peptide (epitope) binding to MHC class I and II molecules, respectively.^22^ Potential cleavage sites against various proteases were predicted using the program PROSPERous (http://propsperous.erc.monash.edu/).^23^

## Results and Discussion

### Biochemical Characterization of Full-Length *A. niger* Amidase 2 Heterologously Produced in *E. coli* (*An*OTA)

The first microbial enzyme reported to effectively hydrolyze OTA was an amidase from *A. niger* referred to as amidohydrolase 2 or amidase 2 to differentiate it from all other *A. niger* amidases reported at that time to which it showed no sequence homology.^9^ This amidase 2 was identified as the OTA-hydrolyzing component of the “Amano lipase” product and subsequently annotated as the first microbial ochratoxinase (OTAse). The 480 amino acid residue amidase 2 from *A. niger* UVK143 (*An*OTA) (100% identical to *A. niger* CBS 513.88 amidase 2) was homologously produced in the *A. niger* AP4 strain.^9^ When the recombinant *An*OTA was purified from the fermentation broth, peptide mass fingerprinting revealed that the extracellular amidase was N-terminally truncated, possessing only 438 amino acid residues, with the first identified residue being Ser-43. Moreover, the authors indicated that this truncation was also observed in purified intracellular *A. niger* amidase.^9^ Additionally, it was described later that the full-length 480 amino acid *An*OTA (referred to as “amidase 2 sig”) also hydrolyzed OTA, similar to the secreted mature 438 amino acid amidase 2 (referred to as “amidase 2 mat”).^24^ It was also observed that the loss of the N-terminal segment containing 42 amino acids of *An*OTA resulted in sensitivity to Ca^2+^ inhibition. These results suggested that although truncated *An*OTA could be used to detoxify OTA, it shows certain limitations due to its sensitivity to divalent metal ions and chelators, widely present in feed additives. In contrast, full-length *An*OTA was described to be much less sensitive to metal ions and chelators, revealing the functional relevance of the truncation of the N-terminal segment.^24^

Considering these results, and that truncated *An*OTA has been partially characterized before, we decided to heterologously produce full-length *An*OTA and perform its enzymatic characterization. To characterize the enzyme, its encoding gene was cloned into the pURI3-Cter expression vector by a ligation-free cloning strategy described previously.^10^ The decision to include the His-tag at the C-terminal end of the protein is twofold: first, the structure of the homo-octameric *An*OTA currently available corresponded to the truncated *An*OTA variant and therefore no structural information from the N-terminal end is known, and second, the presence of the His-tag at the C-terminal end is a priori compatible with the integrity of the oligomer since the tag would protrude toward the solvent.

Hence, the 1.4-kb *An*OTA gene, PCR amplified with oligonucleotides Fw and Rv, was expressed in *E. coli*, and the hyperproduced, C-terminally His-tagged recombinant protein was purified by IMAC, as described in Materials and Methods. In these first approaches for *An*OTA production, we observed a very poor production yield, which agrees well with previous attempts by others to increase the yield by coexpression of the enzyme gene with those coding for molecular chaperones.^25^ Here, we obtained a notable increase in the production yield of the full-length *An*OTA following another approach, namely, *E. coli* codon optimization. This procedure rendered a production yield of 3.72 mg of recombinant protein per liter of culture (Figure S2).

**Figure 1:**
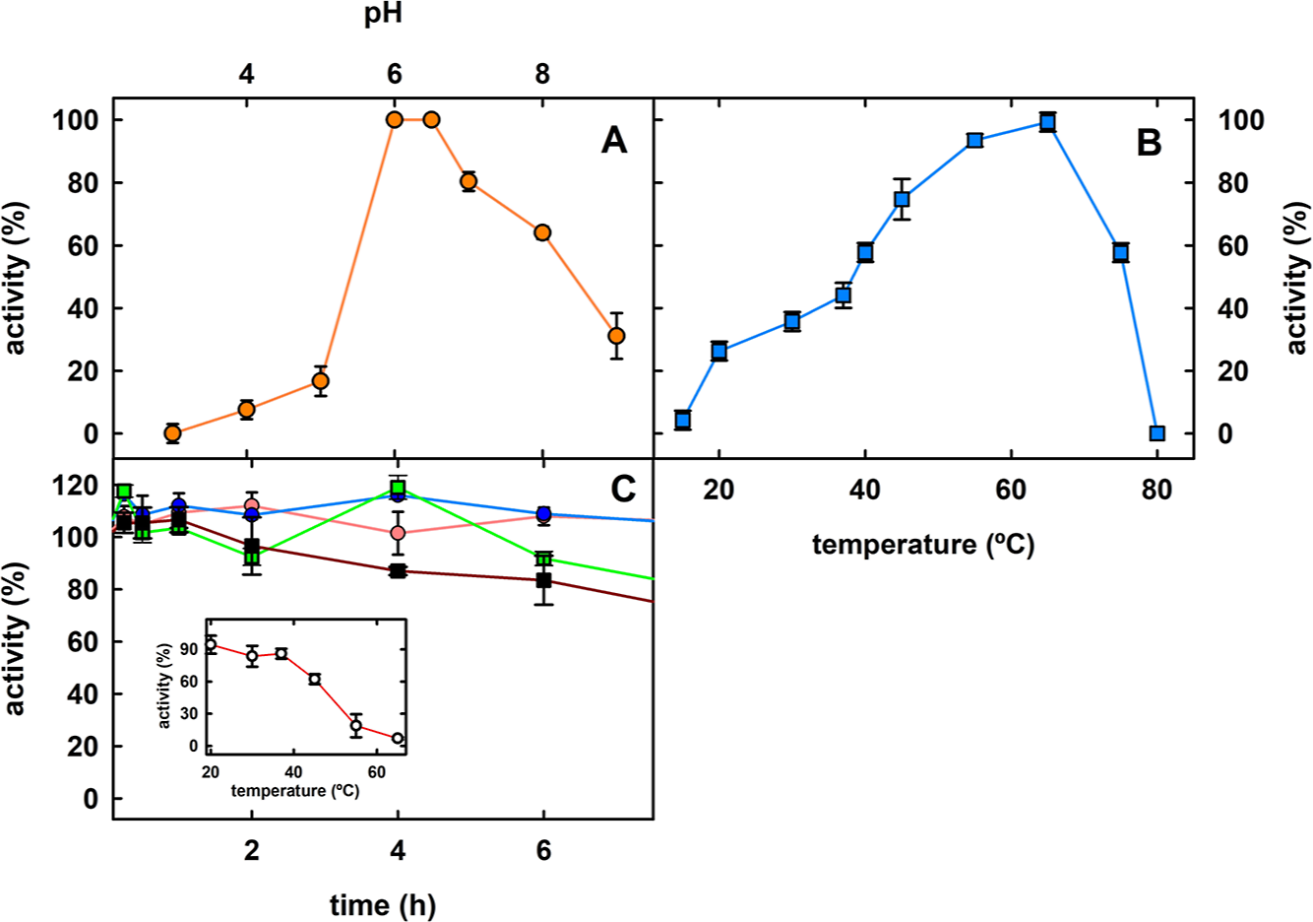
Biochemical properties of *An*OTA from *A. niger* CBS 513.88. (A) *An*OTA pH activity profile. (B) *An*OTA temperature activity profile. (C) *An*OTA thermal stability after incubation at 20 (orange circles and line), 37 (blue circles and line), 55 (green squares and line), and 65 °C (black squares and line), in MOPS buffer (50 mM, 20 mM NaCl, and pH 7) at indicated times. Inset: activity values at 20 h incubation at different temperatures. The mean value and standard error are shown (*n* = 3). The percentage of residual activity was calculated by comparing it to the nonincubated enzyme.

The dependence on pH and temperature of OTA degradation was previously assayed in recombinant *A. niger* amidase 2. The enzyme was active within the pH range between 3.0 and 9.0, with an optimum pH of 7.0.^24^ This same study revealed that recombinant *An*OTA was heat stable since more than 80% of its activity remained after preincubation for 5–30 min at 80 °C.^24^ Additionally, the effect of pH and temperature on truncated *An*OTA has been also described, using the OTA analogue 4MF as a substrate.^9^ Truncated *An*OTA hydrolyzes 4MF optimally at pH 5.6–6.0, and at pH 9.0, approximately 50% of the maximum activity is retained. The optimal temperature for 4MF hydrolysis was determined to be approximately 66 °C. Our results with the full-length *An*OTA, using 4MF as a substrate, revealed pH and temperature dependences similar to those from the truncated protein:^9^ the optimal pH determined here was 6.0–6.5, exhibiting 40% of its maximal activity at pH 9.0 (Figure 1A), and maximal activity at 65 °C, retaining 60% of maximal activity at 75 °C (Figure 1B). However, *An*OTA is fully inactivated after 15 min of incubation at 85 °C, in agreement with the thermostability results described for the truncated *An*OTA.^9^ Thermostability assays performed in this study with *An*OTA revealed that after prolonged incubation (20 h) at 45 °C, the enzyme retained more than 60% of its initial activity and more than 70% at temperatures lower than 45 °C (Figure 1C). Therefore, using 4MF as a substrate, the pH and temperature effects on *An*OTA activity obtained in this study are almost identical to those reported previously for the N-terminal truncated protein. The high optimal temperature shown by *An*OTA, together with its thermostability and the fact that the truncated enzyme has been described to be stable for at least two years without loss of activity,^9^ represents a great advantage for the potential application of *An*OTA in food and feed processing.

The crystal structure of *An*OTA^9^ demonstrated that it belongs to the superfamily of amidohydrolases, in particular, to subtype I of such enzymes, following a classification that considers seven subtypes according to the structural features of their metal(s)-binding centers.^26^ In particular, members from the most common subtype I have two metal ions (*M*_α_ and *M*_β_, respectively) that are coordinated by the side chains of six residues, namely, *M*_α_ is coordinated by two His residues (located in an HXH sequence motif) and an Asp, and *M*_β_ is coordinated by two His residues. In turn, the two metals are bridged by a carboxylated lysine residue, that originated from a post-translational modification. In the members of subtype II, this latter carboxylated lysine is replaced by a glutamate residue, conserving the other protein ligands. Specifically, within the active site of *An*OTA, there are two Zn^2+^ ions, which are coordinated by His111, His113, and Asp378 (M_α_ site) and His287 and His307 (M_β_ site), respectively. The carboxylated Lys246 residue acts as a bridge between them. These same structural features are also observed in the recently reported cryo-EM structure of the homologue enzyme ADH3 from *Stenotrophomonas acidaminiphila*(27) (*Sa*OTA). The participation of the metal-binding center in the catalysis is evidenced in *An*OTA by its complete inactivation with the Zn^2+^-specific chelator 1,10-phenanthroline^9^ and in ADH3 by the complete lack of activity of single-point mutants of residues that participate in the coordination of the metals; this latter analysis concludes that both metals, M_α_ and M_β_, are essential for catalysis.^27^

Within the metalloprotease realm, this type of a bimetallic Zn^2+^ site is defined as cocatalytic, distinct from other sites containing only one Zn^2+^ atom known either as catalytic or structural*.*^28,29^ In agreement with the crystal structure of *An*OTA,^9^ the recombinant, full-length form of the enzyme here studied has Zn^2+^ atoms as deduced from our atomic absorbance analysis, which in turn discards the presence of Co^2+^ that could be derived from the purification protocol. Although the most frequently found metal in cocatalytic sites in metalloproteases is precisely Zn^2+^, there are other cases in which Co^2+^, Mn^2+^, Fe^2+^, and Ni^2+^ have also been identified.^29^

**Figure 2:**
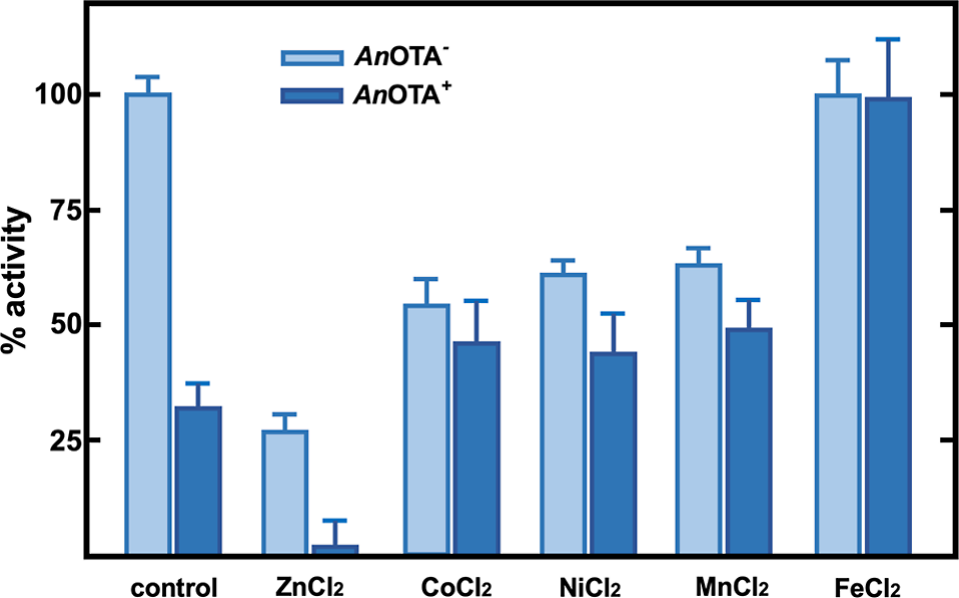
Effects of divalent cations on untreated (*An*OTA^–^; light blue) and EDTA-treated (*An*OTA^+^; dark blue) *An*OTA activity. The relative activity values of *An*OTA^–^ or *An*OTA^+^ are those registered after incubation for 15 min with 1 mM concentration of different divalent cations. The activity of corresponding to *An*OTA^–^ in the absence of added divalent cations was defined as 100%. Light-blue bars correspond to *An*OTA^–^ and dark ones correspond to *An*OTA^+^. The experiments were performed in triplicate. The mean value and standard error are shown.

Under this scenario, we have studied the effect of several metals on *An*OTA activity both with the EDTA-untreated (*An*OTA^–^) and the EDTA-treated (*An*OTA^+^) enzyme. A summary of the obtained results is shown in Figure 2. Regarding the control *An*OTA, we noticed that even treatment with 20 mM EDTA, and subsequent dialysis, does not completely abolish its activity, which supports the idea that *An*OTA exhibits a high affinity for Zn^2+^, in agreement with our atomic absorbance results. Interestingly, the addition of 1 mM Zn^2+^ either to *An*OTA^–^ or *An*OTA^+^ results in a strong inhibition of its activity. Although counterintuitive it may be, the lack of activity in zinc enzymes in the presence of an excess of this metal (here, 1 mM) has been described before.^28−30^ In this regard, previous studies on *E. coli* aminopeptidase P have revealed a third Zn-binding site, also within the active site of the enzyme, close to the other two catalytic Zn^2+^ atoms,^31^ whose occupancy causes metal inhibition. Similar results have been obtained for the Mn^2+^ inhibition of the aminopeptidase P from *Pseudomonas aeruginosa.*(32)

The results obtained with 1 mM Co^2+^ when added to *An*OTA^–^ can be easily explained in terms of a much less efficient inhibition than that exerted by Zn^2+^, most probably resulting from the low-affinity binding of Co^2+^ to a putative third, inhibitory metal-binding site. The activity level observed when Co^2+^ is added to *An*OTA^+^ would support the latter low-affinity binding to the third metal-binding site, and importantly, also that Co^2+^ would replace the removed Zn^2+^ atom(s) from the cocatalytic site. Since, essentially, the same results are obtained with Ni^2+^ and Mn^2+^, a similar hypothesis can be proposed for these metals. In contrast to Co^2+^, Ni^2+^, and Mn^2+^, the 100% activity level observed for either *An*OTA^–^ or *An*OTA^+^ when treated with iron would indicate that this metal binds to the cocatalytic site but not to the third inhibitory site, and therefore no inhibition would be exerted.

**Figure 3:**
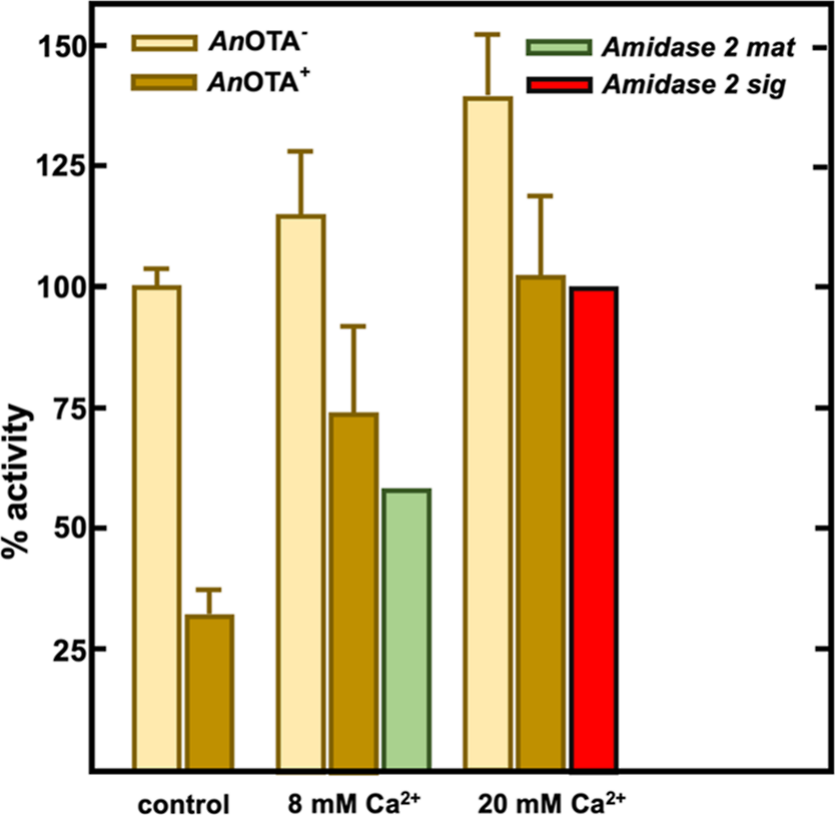
Effect of 8 and 20 mM CaCl_2_ on *An*OTA^–^ and *An*OTA^+^. Proteins were preincubated with the corresponding additives during 15 min at room temperature before the reaction with 4MF as a substrate. The error bars represent the standard deviation estimated from three independent assays. Light-brown bars represent *An*OTA^–^ (EDTA-untreated *An*OTA), and dark-brown bars represent *An*OTA^+^. Green bar represents the activity of amidase 2 mat (truncated form of *An*OTA), and the red one represents the activity of amidase 2 sig (*An*OTA). These two latter results are from Yu et al. (2015).^24^ The activity exhibited by native untreated *An*OTA in the absence of additive was defined as 100%.

As we have indicated above, Yu et al. reported that *An*OTA (or amidase 2 sig) exhibits lower Ca^2+^ inhibition sensitivity as compared to the N-terminal truncated protein (amidase 2 mat).^24^ In turn, these authors also showed that the activity of amidase 2 mat was 40% inhibited by 8 mM CaCl_2_, while *An*OTA was not appreciably inhibited neither by 20 mM EDTA nor 20 mM Ca^2+^.^24^ In agreement with these results, we observed that the activities of both *An*OTA^–^ or *An*OTA^+^ enzymes did not decrease but increased 14 and 34%, respectively, upon preincubation with 8 mM CaCl_2_ (Figure 3). Therefore, our results confirmed the low sensitivity of full-length *An*OTA and also its suitability for OTA detoxification in food or feed additives containing a chelator. Citric acid or other organic acids such as propionic acid are widely used as feed additives and can chelate divalent ions;^24^ therefore, the use of an enzyme, such as the full-length *An*OTA, less sensitive to divalent metal ions and to chelators will be strongly recommended.

### Analysis of the Substrate Specificity of *An*OTA

As indicated above, determination of the substrate profile of an enzyme to be used in detoxification processes in complex, protein-rich milieus is a must, since degradation of off-target compounds must be avoided. This is particularly a concern for the members of the amidohydrolase superfamily that despite sharing a highly conserved overall protein subunit architecture, they show disparate substrate specificities due to differences in structural elements decorating their substrate-binding sites. Hence, within the closest structural homologues of *An*OTA (see Table 1 and Figure S3), the three enzymes isolated from an environmental sample from the Sargasso Sea, Sgx9359b,^33^ Sgx9355e,^34^ and Sgx9260c,^15^ whose subunit structures superimpose almost perfectly to *An*OTA, show markedly different substrate specificities, namely, Sgx9359b catalyzes the hydrolysis of L-Xxx-l-Arg dipeptides (and *N*-acetyl and *N*-formyl derivatives), Sgx9355e hydrolyzes dipeptides with a C-terminal hydrophobic amino acid (Ile, Leu, Phe, Tyr, Val, Met, and Trp), although also accepts a C-terminal Thr, and Sgx9260c hydrolyzes Gly-l-Pro and l-Ala-l-Pro (and *N*-acyl derivatives of l-Pro).

**Table 1:** Closest Structural Homologues of *An*OTA and Substrate Specificity

PDB	biological source	activity (reference)	s.i^a^	rmsd^b^
3DUG	Sgx9359b^c^	l-Arg carboxypeptidase^33^	33	2.7
2QS8	Sgx9355b^c^	Xaa-Hyd^d^ dipeptidase^34^	28	2.5
3MKV	Sgx9360b^c^	Gly/Ala/N-acyl-Pro dipeptidase^15^	29	2.5
3MTW	Caulobacter crescentus Cc2672	l-Arg/Lys carboxypeptidase^33^	30	2.8
8THS	*Stenotrophomonas acidaminiphila*	amidohydrolase^27^	30	2.5

a s.i.: amino acid sequence identity (%).

b rmsd: root-mean-square deviation (Å).

c Protein whose DNA has been isolated from the Sargasso Sea.

d Hyd: hydrophobic, C-terminal amino acid.

Cc2672 from *Caulobacter crescentus* CB15 hydrolyzes L-Xaa-l-Arg/Lys dipeptides,^33^ and the amidohydrolase from *Stenotrophomonas* sp. CW117 is known to hydrolyze OTA. However, no further data have been reported about its substrate profile. Other structurally more distant homologues of *An*OTA such as peptidases belonging to the M38 family of enzymes from the MEROPS peptidase database^35^ show a wide spectrum of specificities, such as isoaspartyl dipeptidase,^36^ dihydropyrimidinase,^37^ guanine deaminase,^38^ urease,^39^ etc.

**Figure 4:**
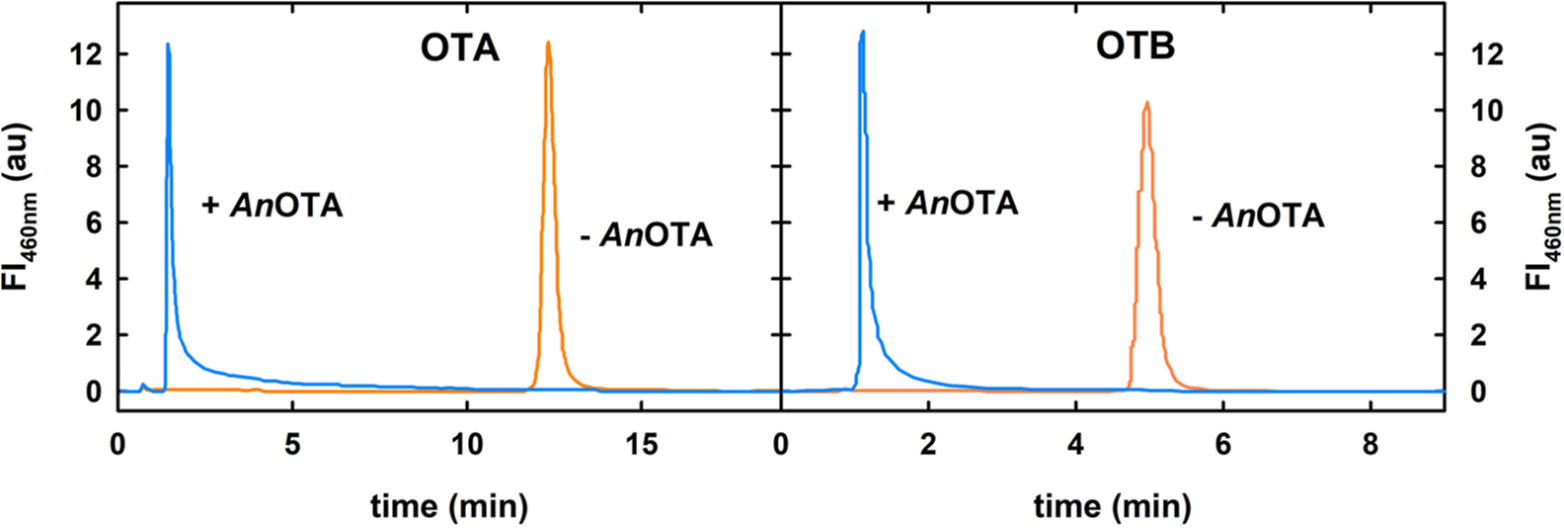
*An*OTA activity on OTA (left) and OTB (right) mycotoxins. The HPLC chromatograms of OTA and OTB (5 μM) incubated at 37 °C for 16 h in the presence of *An*OTA are shown in blue lines. Control reactions without *An*OTA are shown in orange. The fluorescence wavelengths were 330 nm for excitation and 460 nm for emission.

As far as we know, no results about the substrate profile of *An*OTA have been reported apart from those described by Dobritzsch et al. that revealed hydrolysis of 4MF, l-Phe-l-Tyr, l-Arg-l-Phe, and hippuryl-l-Phe,^9^ which supports a preference for an aromatic C-terminal residue in model compounds with one amide bond. To characterize the substrate profile of *An*OTA in further detail, we have used a library of 17 *N*-benzyloxycarbonyl (or carbobenzyloxy) amino acid derivatives, ten *N*-acetyl derivatives of l-amino acids, two *N*-benzoyl-glycyl amino acid derivatives (or hippuryl-amino acids), and two L-dipeptides (Figure S4). However, before proceeding with this set of potential substrates, we first confirmed the hydrolytic activity of *An*OTA against OTA, and also OTB, and second, we also tested potential endopeptidase activity against BSA. As expected, we observed complete hydrolysis of OTA and OTB by full-length *An*OTA after overnight incubation (Figure 4), but no endopeptidase activity, as deduced by the absence of fragmentation products in an SDS-PAGE gel (data not shown).

**Figure 5:**
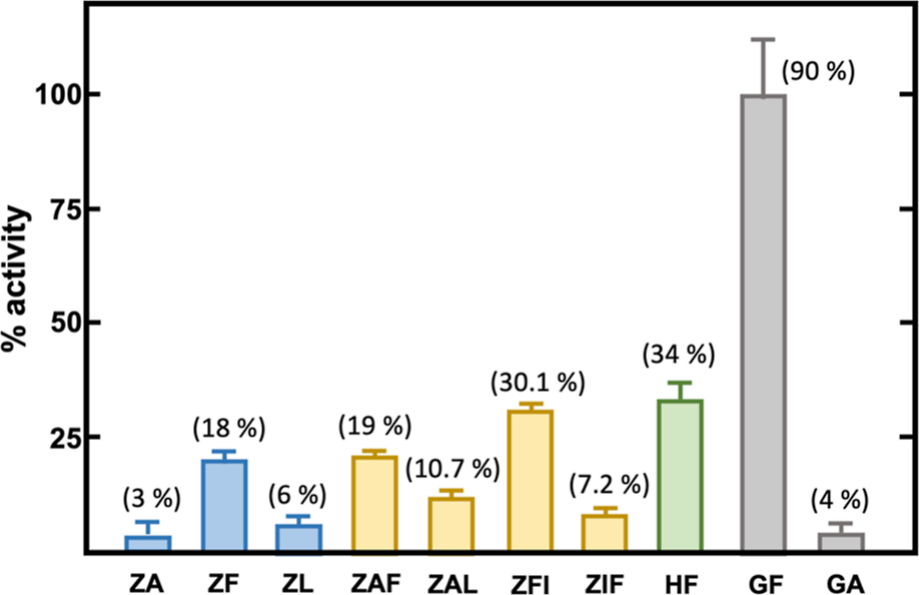
Specificity profile of *An*OTA. The height of the bars represents the enzymatic activity level of *An*OTA against different substrates, normalized to the maximum observed activity (against glycyl-phenylalanine; GF). Only the results from hydrolyzed substrates are shown. Each color corresponds to a different family of substrates (see Figure S4): blue, *N*-benzyloxycarbonyl amino acid derivatives with one amide bond; light brown, *N*-benzyloxycarbonyl amino acid derivatives with two amide bonds; green, hippuryl-amino acids; and gray, l-dipeptides. ZA: *N*-benzyloxycarbonyl-l-alanine; ZF: *N*-benzyloxycarbonyl-l-phenylalanine; and ZL: *N*-benzyloxycarbonyl-l-leucine. ZAF: *N*-benzyloxycarbonyl-l-alanyl-l-phenylalanine; ZAL: *N*-benzyloxycarbonyl-l-alanyl-l-leucine; and ZFI: *N*-benzyloxycarbonyl-l-phenylalanine-l-isoleucine. HF: hippuryl-l-phenylalanine. GF: l-glycyl-phenylalanine. GA: l-glycyl-alanine. The error bars represent the standard deviation estimated from the three independent assays. The percentage of hydrolyzed compounds is indicated on top of each bar (initial concentration: 1 mM).

The results of the hydrolytic activity of *An*OTA against the set of compounds mentioned above reveal that the dipeptide GF is the most efficiently hydrolyzed substrate. Also, only three *N*-benzyloxycarbonyl derivatives containing one amide bond out of 12 are hydrolyzed: ZF > ZL ∼ ZA (Figure 5), and only four *N*-benzyloxycarbonyl derivatives containing two amide bonds out of five are hydrolyzed, namely, ZFI > ZAF > ZAL > ZIF (Figure 5). Finally, regarding the hippuryl compounds, only HF is hydrolyzed. From these results, it can be concluded that (i) hydrolysis by *An*OTA is exclusively observed against compounds containing a C-terminal Phe or aliphatic amino acid (Ala, Leu, and Ile); (ii) *An*OTA hydrolyzes *N*-benzyloxycarbonyl derivatives containing either one or two potentially scissile amide bonds; (iii) *N*-acetyl-l-amino acids are not hydrolyzed by *An*OTA, irrespective of the amino acid residue; hence, most probably, *An*OTA is not an *N*-acetyl amidohydrolase; and (iv) compounds with a C-terminal polar or charged side chain are not hydrolyzed.

Regarding the *N*-benzyloxycarbonyl derivatives containing two potentially scissile amide bonds, it is worth noting that the above results do not permit a response neither to the question of how many amide bonds are hydrolyzed nor to the order of hydrolysis in case both amide bonds are hydrolyzed. Although the absence of endopeptidase for *An*OTA suggests that the unique scissile amide bond of these *N*-benzyloxycarbonyl derivatives is the C-terminal one, we tackle these questions from a structural perspective using molecular docking simulations following the same experimental approach as in our previous studies.^13,40^ Previous to these studies, and to validate our approach, we proceeded to dock the OTA molecule into the binding site of *Sa*OTA and compared the results with the cryoEM structure of such a complex.^28^ As can be observed in Figure S5, the obtained results are in excellent agreement with the experimental structure (PDB entry: 8IHS), since the docked OTA molecule superimposes almost perfectly with the experimental one, revealing the same pattern of interactions with the amino acid side chains of *Sa*OTA and the Zn^2+^ ions.

**Figure 6:**
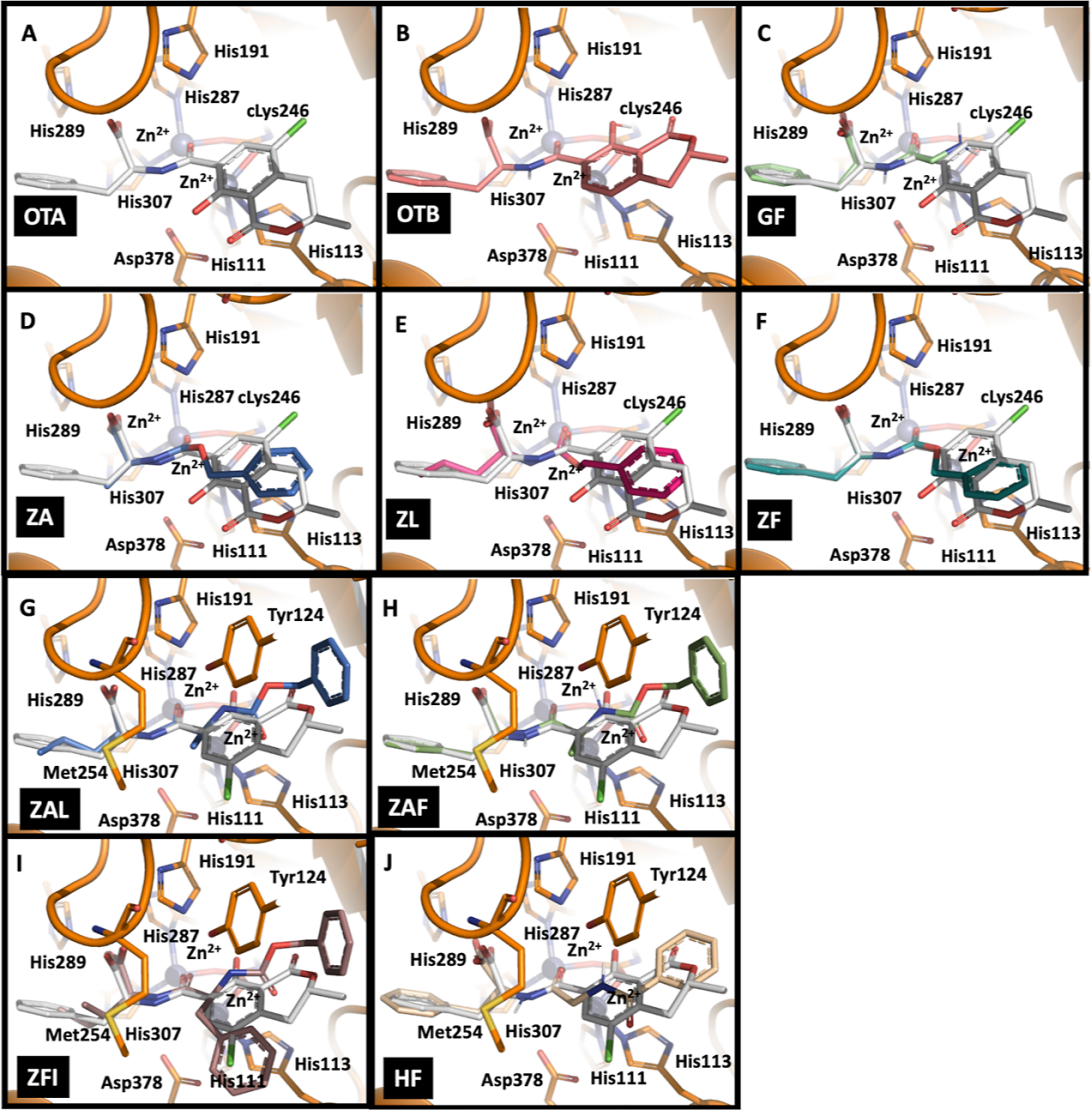
*In silico* molecular docking of OTA (panel A), OTB (panel B), and the substrates hydrolyzed by *An*OTA into the active site of the enzyme. Each panel represents the binding mode for the different substrates, namely, GF, l-glycyl-phenylalanine (C); ZA, *N*-benzyloxycarbonyl-l-alanine (D); ZL, *N*-benzyloxycarbonyl-l-leucine (E); ZF, *N*-benzyloxycarbonyl-l-phenylalanine (F); ZAL, *N*-benzyloxycarbonyl-l-alanyl-l-leucine (G); ZAF, *N*-benzyloxycarbonyl-l-alanyl-l-phenylalanine (H); ZFI, *N*-benzyloxycarbonyl-l-phenylalanine-l-isoleucine (I); and HF, hippuryl-l-phenylalanine (J). Important amino acid side chains are shown in sticks, and the Zn^2+^ ions are shown as spheres.

Molecular docking of OTA into the active site of *An*OTA revealed a similar binding mode as in *Sa*OTA (Figure 6A), which entails the following interactions: (i) the C-terminal, Phe aromatic ring of OTA is located in an aliphatic pocket formed by the side chains of Val253, Val332, Ile333, and Leu355; (ii) the negatively charged carboxy moiety would interact favorably with the nearby side chains of His191, His287, and His289; (iii) the OTA carbonyl oxygen of the scissile amide bond would point toward the Zn^2+^ (M_β_ site), which is consistent with the catalytic mechanism of hydrolysis of metallocarboxypeptidases;^13,41^ and (iv) the N-terminal, 5-chloro-8-hydroxy-3-methyl-1-oxo-7-isochromanyl ring of OTA (ISO) would establish aromatic stacking interactions with the imidazole side chain of His113.

Interestingly, docking of OTB, which is the nonchlorinated version of OTA, reveals that its N-terminal 8-hydroxy-3-methyl-1-oxo-7-isochromanyl ring is rotated 180° when compared to OTA (Figure 6B), revealing a different binding mode of the N-terminal end. This unexpected result may provide the structural basis underlying the higher efficiency of hydrolysis of OTA vs OTB by *An*OTA, as has been reported previously.^24^

The molecular docking results obtained with the dipeptide GF reveal that this substrate binds as the structurally equivalent C-terminal end of OTA (Figure 6C). Taking into account the structure of this substrate, this result suggests that the built-up of the interactions between the C-terminal end of the substrates and the aliphatic pocket of *An*OTA is the main driving force for yielding a catalytically productive binding; in fact, the location of the C-terminal end of substrates in this pocket is a shared feature for all substrates studied here. Thus, as expected, the results obtained with the *N*-benzyloxycarbonyl-l-amino acid substrates ZA, ZL, and ZF (Figure 6F) reveal binding modes which are also consistent with the binding of OTA. Specifically, the C-terminal amino acid side chain is located in the aliphatic pocket with their N-terminal carbobenzyloxy moieties occupying equivalent positions as that of ISO. Importantly, in all cases, the scissile amide bond occupies the same position with the same orientation. Regarding the *N*-benzyloxycarbonyl-l-amino acid substrates containing two amide bonds ZAL, ZAF, and ZFI, the docking results indicate that the binding site of *An*OTA accommodates these larger molecules (Figure 6I). Whereas the C-terminal amino acid side chain is housed in the aliphatic pocket, the N-terminal aromatic ring of the *N*-benzyloxycarbonyl moiety would establish favorable stacking interactions with the side chain of Tyr124; finally, the arrangement of the side chain of the intermediate residue of the substrate (Ala in ZAL or ZAF, and Phe in ZFI) would be constrained by the bulky side chain of Met254. Finally, similar conclusions can be deduced from the docking of the substrate HF (Figure 6J): its C-terminal end is situated within the aliphatic pocket, as expected. Interestingly, no hydrolytic activity is observed against the other hippuryl derivative studied here (hippuryl-arginine, HR) which bears a positively charged C-terminal amino acid side chain. As expected, no pose compatible with the catalytic mechanism of hydrolysis is obtained in the docking assays, indicating that the positively charged side chain cannot be stabilized within the aliphatic pocket.

These in silico results provide the structural details revealing that *An*OTA only hydrolyzes the C-terminal amide bond of the model substrates. In this regard, it has not escaped our notice that since hydrolysis of the C-terminal residue of ZAL, ZAF, or ZFI produces ZA and ZF, respectively, which in turn are substrates for *An*OTA, they could be also hydrolyzed. Nevertheless, we consider that the contribution of this second hydrolysis reaction is negligible since ZA and ZF concentrations (resulting from the hydrolysis of ZAL, ZAF, and ZFI, respectively) are much lower that ZAL, ZAF, and ZFI in our experimental conditions.

Hence, our studies provide a plausible scenario about the structural bases of the mycotoxin- and substrate-binding modes supporting the notion that *An*OTA is a carboxypeptidase that shows a marked preference for C-terminal Leu, Phe, or Ala residues. The *An*OTA binding site is an open structure that permits the binding of large substrates such as ZAL, ZAF, ZFI and ZIF, which in turn explains previous results reporting the hydrolysis of the dipeptide *N*-benzyloxycarbonyl-glycine-phenylalanine, tripeptide *N*-benzyloxycarbonyl-glycine-phenylalanine-phenylalanine, and pentapeptide *N*-benzyloxycarbonyl-glycine-leucine-glycine-phenylalanine.^25^ This ability to accommodate a wide range of substrates, or promiscuity, is especially relevant in the case of amidohydrolases which can hydrolyze a wide range of substrates. Therefore, it can be suggested that in OTA-producing fungi, it is likely that the physiological substrate of *An*OTA, and close homologues, is ochratoxins. In this sense, it has been described that *An*OTA-like enzymes from OTA-producing fungi showed more favorable substrate-binding pockets to accommodate OTA and OTB than similar enzymes from other nonproducer fungi.^42^

The OTA-hydrolyzing activity of *An*OTA, and probably that of other homologues from OTA-producing fungi, together with its marked preference for C-terminal Phe, Ala, Leu, and Ile residues of *An*OTA represent an advantage for the use of these enzymes in detoxification processes since the production of unwanted byproducts in protein-rich food substrates is minimized, preventing the occurrence of relevant changes in the organoleptic and nutritional characteristics of the food substrate.

### Application of *An*OTA in Plant-Based Beverages for OTA Detoxification

The emergence of plant-based beverages as a nondairy alternative offers a new option due to environmental and ethical concerns, as well as lactose intolerance and casein allergies. However, raw materials employed in the elaboration of these beverages are susceptible to mycotoxin contamination. For this reason, in this work, we carried out OTA-detoxification assays by *An*OTA in oat, soy, and almond beverages.

**Figure 7:**
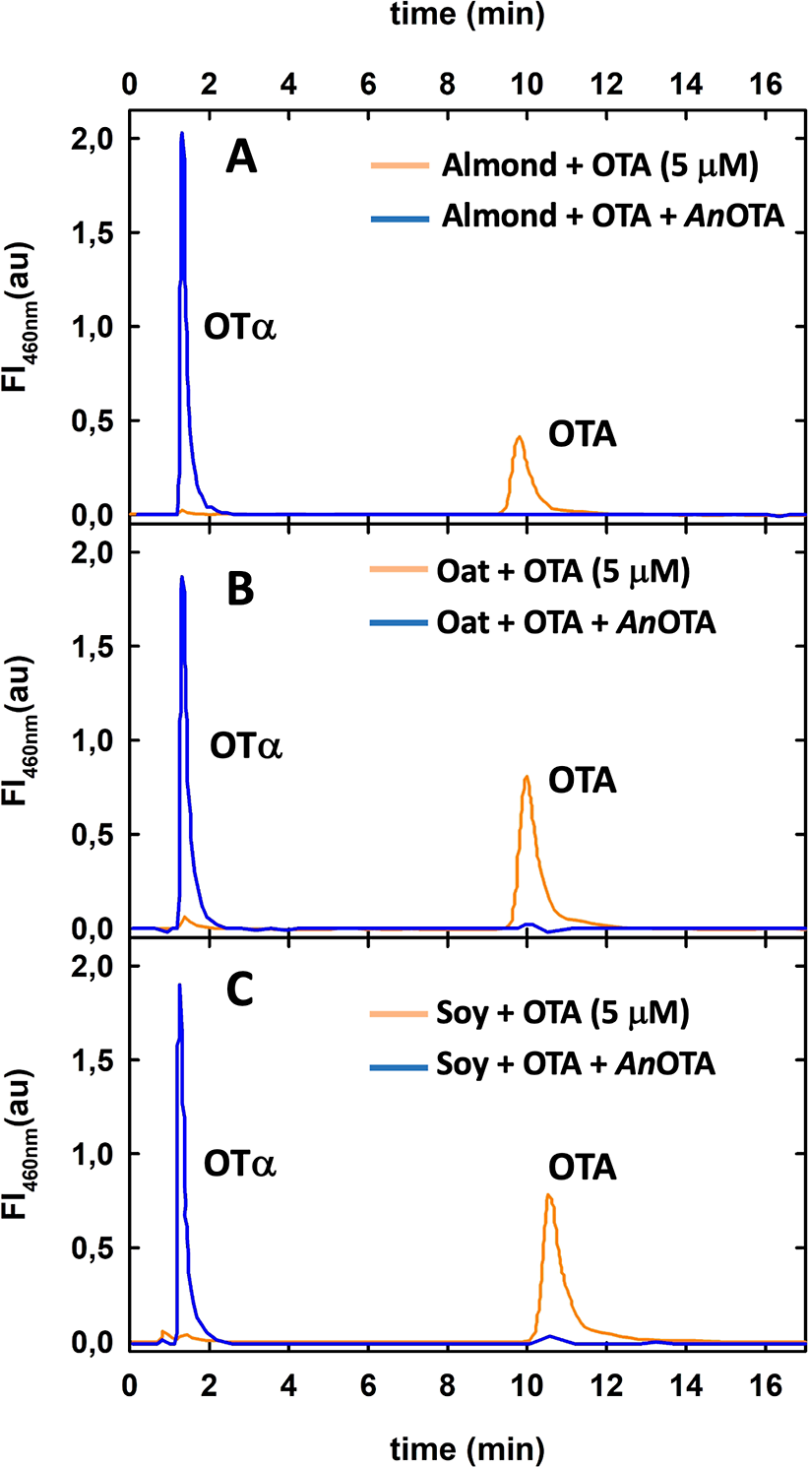
*An*OTA activity on OTA containing plant-food beverages. HPLC chromatograms of (A) almond, (B) oat, and (C) soy commercial beverages containing 5 μM OTA, without *An*OTA (orange traces) and incubated with *An*OTA at 20 °C for 4 h are shown (blue traces). The fluorescence wavelengths were 340 nm for excitation and 460 nm for emission.

Plant-based beverages contain proteins, lipids, dietary fiber, minerals, and vitamins. Enzymatic hydrolysis (such as amylase, protease, cellulase, and pectinase) is a specific and effective method to hydrolyze vegetable components to convert some of them into easily absorbed nutrients like glucose, maltose, amino acids, and soluble phenolics. As reported in their labels, the three beverages assayed here possessed a diverse biochemical composition. To know if these plant-based beverages possessed adequate properties for *An*OTA activity, their pH was determined. All beverages assayed possessed a close to neutral pH, ranging from a pH of 7.16 in the soy beverage to a pH of 7.84 in the almond beverage. The optimal pH for *An*OTA activity is 6.0–6.5 (Figure 1A), although almost 60% of its maximal activity was found at pH 8.0. Concerning temperature, plant-based beverages containing *An*OTA were incubated at 20 and 37 °C. Incubation at a temperature close to room temperature (20 °C) was chosen as it is an economically affordable temperature since at this temperature, *An*OTA could exert its activity during beverage storage. In spite that the optimal temperature for *An*OTA activity is high (65 °C), at 20 and 37 °C, this enzyme exhibited 30 and 40% of its maximal activity (Figure 1B). Therefore, at the pH of the beverage assayed and the incubation temperatures used in the study, *An*OTA should remain active (Figure 1A). Figure 7 shows that OTA was fully hydrolyzed in all beverages incubated during 4 h at 20 °C; identical results were obtained at 37 °C incubation (data not shown). These results indicate that *An*OTA can be used efficiently to eliminate OTA from plant-food beverages, even at storage conditions.

Activity assays performed in this study revealed that *An*OTA does not possess endopeptidase activity on BSA. To confirm the absence of endopeptidase activity in these assays, plant-food beverages were incubated for 18 h in the presence of *An*OTA at 37 °C, since at this temperature, enzyme activity is higher than at 20 °C. After incubation, proteins in beverage samples were analyzed on SDS-PAGE gels. Previously, protein content was spectrophotometrically determined. The results indicated that soy beverage possessed the highest protein content (88.19 mg/mL), followed by almond (40.34 mg/mL) and oat (34.68 mg/mL) beverages. As shown in Figure S6, the protein profile of the *An*OTA-treated beverages presented identical protein profiles to the nontreated beverages. Most of the protein bands from oat beverage correspond to proteins lower than 45 kDa; however, almond and soy beverages also possess proteins having high molecular size. In any case, *An*OTA treatment did not produce an evident variation in the protein profile of the beverage proteins.

**Figure 8:**
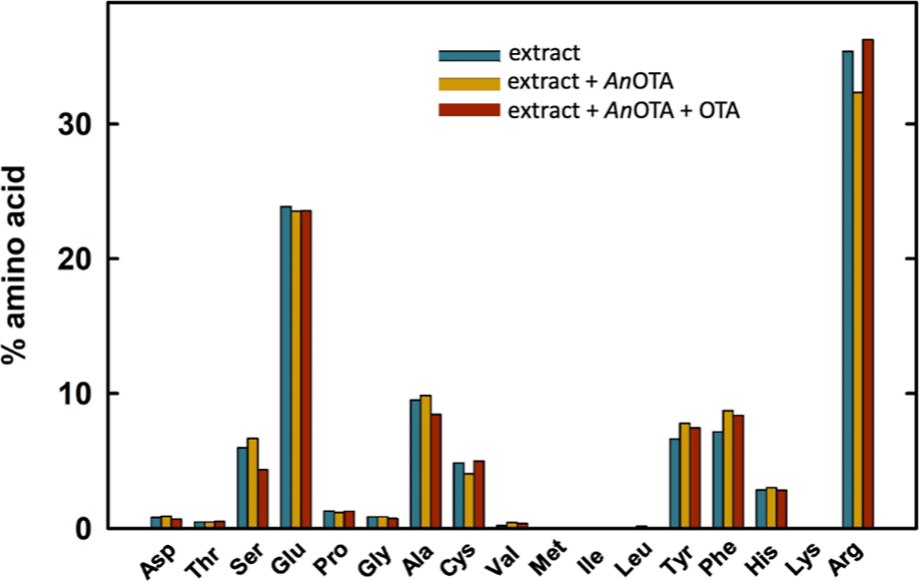
Free amino acid composition of soy commercial beverages. The relative amount of each amino acid is shown in different conditions: control without *An*OTA and OTA (green); with *An*OTA (orange); and with *An*OTA and OTA (red). The reactions were performed with the soy beverages by adding OTA at 5 μM final concentration (red) and using 5 μg of the *An*OTA enzyme (orange and red). Reactions were incubated at 20 °C for 4 h.

In addition to the study of the potential hydrolytic effect of *An*OTA on the plant beverages by analyzing the pattern of protein bands in SDS-PAGE, the free amino acid composition of *An*OTA-treated and untreated soy beverage was also studied (Figure 8). The obtained results indicate that the amino acid composition does not vary significantly upon addition of *An*OTA, suggesting that the unwanted hydrolytic effect on the proteins from the beverage is negligible, both in the absence and in the presence of OTA. More rigorously, we used as an estimation of the overall uncertainty in the values of the relative amount of the amino acids the mean of the standard deviations (s.d.) of the data for the amino acids that cannot derive from the catalytic activity of *An*OTA, namely, polar or charged residues (Asp, Glu, Lys, Arg, Ser, and His). The calculated overall s.d. is 0.9. In turn, the s.d. values for Phe and Tyr were 0.8 and 0.6, respectively, revealing that the variation of the values for these two latter amino acids fall within the instrumental uncertainty. Therefore, we conclude that the addition of *An*OTA to plant-food beverages, with or without OTA, does not yield unwanted byproducts originated from the hydrolysis of plant proteins. These results support our claim that the OTA detoxification with *An*OTA may be a promising approach to eliminate this mycotoxin effectively, without modifying the beverage characteristics.

### Computational Predictions of *An*OTA In Vivo Characteristics

Like other proteins, once ingested, enzymes are generally easily broken down into their constituent amino acids that are indistinguishable from other food molecules. In general, ingestion of microbial enzymes is not likely to be of concern regarding food allergies.^43^ However, allergenicity, antigenicity, and immunogenicity of food proteins are crucial aspects associated with the widespread usage of new foods and additives. As OTA-degrading enzymes have a potential application in the food industry, the potential allergenicity and antigenicity of *An*OTA were predicted in silico. In this regard, the prediction of allergenic proteins has become very relevant nowadays due to the use of enzymes in foods, therapeutic compounds, and biopharmaceuticals. Currently, allergen prediction methods are heavily used by the scientific community in designing proteins with low allergenicity. As a first approach to studying the allergenicity of *An*OTA, the AllerTOP v.2 and AlgPred programs were used.^19,20^ The results obtained with both tools indicate that *An*OTA is a nonallergenic protein. Here, it is important to remark that the criteria used for each program to define *An*OTA as nonallergenic are different, and therefore, the overall results are not redundant. Thus, whereas AllerTOP v.2 predicts protein allergenicity based on known allergenic and nonallergenic protein sequences, AlgPred combines three methods: (i) a BLAST search to identify allergens based on their similarity to known allergens; (ii) prediction of IgE epitopes, and (iii) *MOTIF scanning* search for conserved motifs in the protein that are also present in allergenic proteins.

Immunoinformatics can also aid in the discovery of immunogenicity prediction. The possibility of an immune response toward *An*OTA was evaluated by the VaxiJen program.^21^ This program is a widely used method to distinguish between immunogens and nonimmunogens among proteins from different origins. In agreement with the above results, VaxiJen assigned a low antigenicity for *An*OTA classifying it as a non-antigen. The prediction of epitope binding to major histocompatibility complex MHC class I and II molecules was made with NetMHCcons and NetMHCIIpan, respectively.^22^ Using the default settings of NetMHCIIpan and considering HLA supertype representatives (the most frequent alleles of HLA A/B), 16 potential epitopes with a length of 15 amino acids were identified in *An*OTA as strong binders to MHC class II. Additional 49 weak binders were also identified to MHC class II. The detected number of epitopes for MHC class I was lower in both forms, being identified only 4 strong binders and 8 weak binders.

Finally, we used the PROSPERous program^23^ to predict potential substrate cleavage sites for 90 proteases. Of these proteases, 51 were of human origin including aspartic protease, cysteine protease, metalloprotease, and serine protease. According to PROSPERous, *An*OTA would be sensitive to multiple proteases as eighty-four cleavage sites were found. Thus, *An*OTA may be sensitive to cathepsin K as a cysteine protease, various matrix metalloproteases, and some serine proteases, including elastase, cathepsin G, and glutamyl peptidase I.

Mycotoxin contamination can cause huge economic losses to global agriculture. The enzymatic biodetoxification of mycotoxins is a suitable strategy due to its high efficiency, strong specificity, and no damage to nutrients. However, these advantages are highly dependent on the biochemical characteristics of the selected enzyme. In this study, *An*OTA peptidase from *A. niger*, generally regarded as a safe organism, has been characterized. The pH and temperature profiles exhibited by *An*OTA, together with its thermostability, make this enzyme appropriate for its use in technological processes. The enzyme exhibited strong specificity toward substrates containing a C-terminal Phe or aliphatic residue, which is a desirable feature since it minimizes potential damage to nutrients. The structural basis for this specificity has been studied by molecular docking simulations, which have revealed the pattern of interactions between the substrates and the amino acid side chains within the *An*OTA active site. Moreover, computationally predicted characteristics indicated that *An*OTA is a nonallergenic, nonantigenic, and low immunogenic protein, which can be cleaved by human proteases. All these in vitro and in silico results support the suitability of *An*OTA for its use for OTA detoxification in food processing and animal feed. However, further studies are needed to decipher the remaining knowledge gap about *An*OTA efficiency in vivo.

## Supporting Information Available

Scheme of the hydrolysis of the amide bond of OTA, SDS-PAGE analysis of recombinant *An*OTA from *A. niger* CBS 513.88, structural comparison of the *An*OTA subunit with those from its close structural homologues, structures of the substrates used in our study classified into five families and also those of OTA and 4MF, docking of OTA into the cryoEM 3D structure of the *An*OTA homologue enzyme ADH3 from *S. acidaminiphila* (*Sa*OTA) and comparison with the experimentally determined structure of the mycotoxin, and SDS-PAGE analysis of plant-based beverages containing OTA (5 μM) incubated with *An*OTA at 37 °C for 18 h (PDF)
