# Supplementary material for: Aspergillus niger Ochratoxinase Is a Highly Specific, Metal-Dependent Amidohydrolase Suitable for OTA Biodetoxification in Food and Feed
Source: J Agric Food Chem. 2024 Aug 7;72(33):18658–69. doi: 10.1021/acs.jafc.4c02944 (PMC11342369; doi:10.1021/acs.jafc.4c02944)
Supplement: Supplementary file 1 — jf4c02944_si_001.pdf [file jf4c02944_si_001.pdf]

Supporting Information

Aspergillus niger ochratoxinase is a highly specific, metal-dependent amidohydrolase suitable for OTA biodetoxification in food and feed

ANA SÁNCHEZ-ARROYO [†], LAURA PLAZA-VINUESA [†], BLANCA DE LAS RIVAS [†], JOSÉ MIGUEL MANCHEÑO[*, ‡], ROSARIO MUÑOZ [*,†]

[†] Institute of Food Science, Technology and Nutrition (ICTAN), CSIC, José Antonio Novais 6, 28040 Madrid, Spain

[‡] Department of Crystallography and Structural Biology, Institute of Physical Chemistry Blas Cabrera (IQF), CSIC, Serrano 119, 28006 Madrid, Spain

*Corresponding authors at: Bacterial Biotechnology Laboratory, Institute of Food Science, Technology and Nutrition (ICTAN), CSIC, José Antonio Novais 6, 28040 Madrid, Spain; E-mail address: r.munoz@csic.es (R. Muñoz), and Department of Crystallography and Structural Biology, Institute of Physical Chemistry Blas Cabrera, CSIC, Serrano 119, 28006 Madrid, Spain; E-mail address: jm.mancheno@csic.es (J.M. Mancheño)

Figure S1

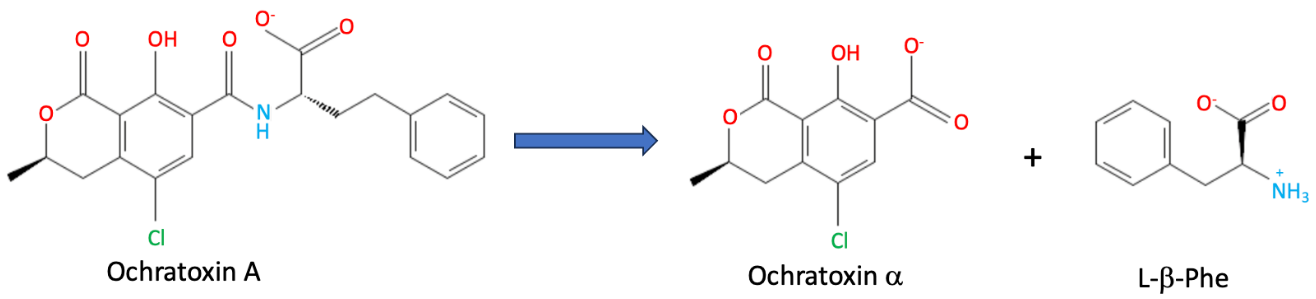

Figure S1. Scheme of the hydrolysis of the amide bond of Ochratoxin A, resulting in the formation of the innocuous products Ochratoxin α and L-β-phenylalanine.

Figure S2

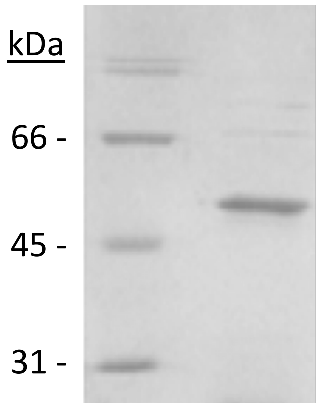

Figure S2. SDS-PAGE analysis of recombinant *An*OTA from *A. niger* CBS 513.88. The 12.5% gel was stained with Coomassie blue. Molecular mass markers are located on the left (SDS-PAGE standards, Bio-Rad).

Figure S3

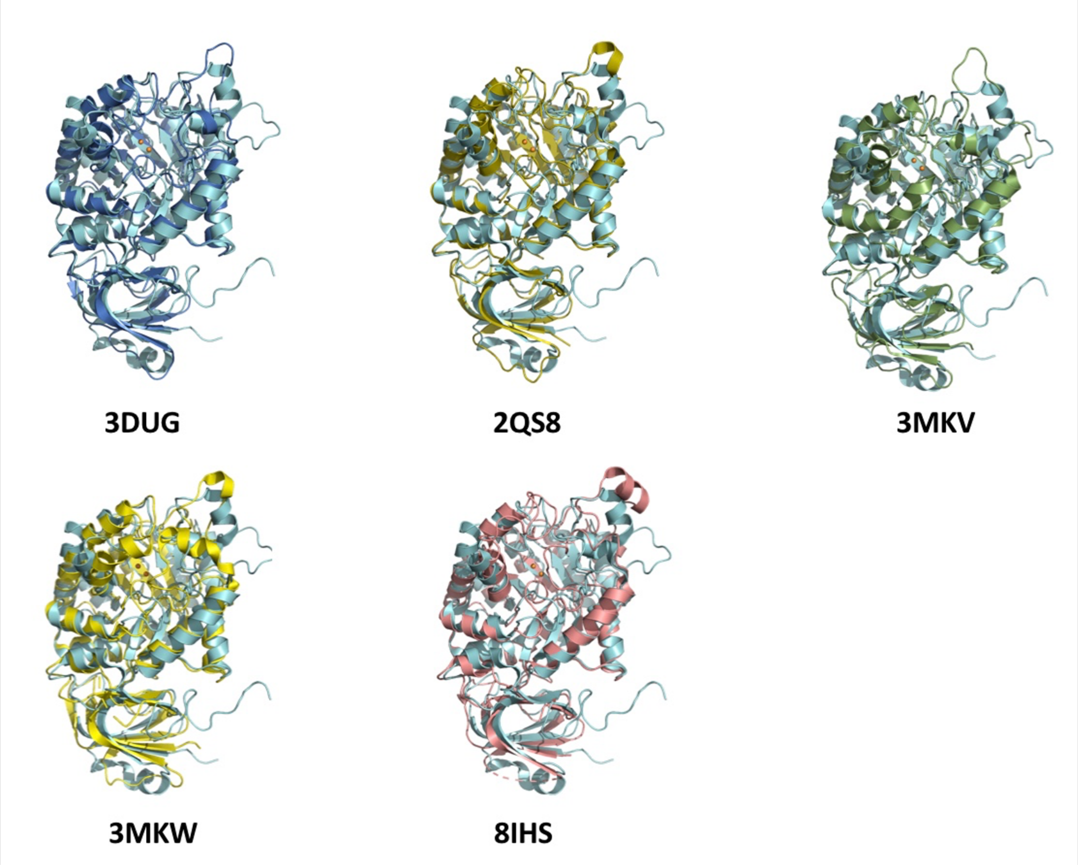

Figure S3. Structural comparison of the *An*OTA subunit with those from its close structural homologs. The crystal structure of *An*OTA (*cyan ribbon*) is superimposed onto those from the close homologs. Pairwise 3D alignments were performed with FATCAT (https://fatcat.godziklab.org/). See also Table 1.

Figure S4

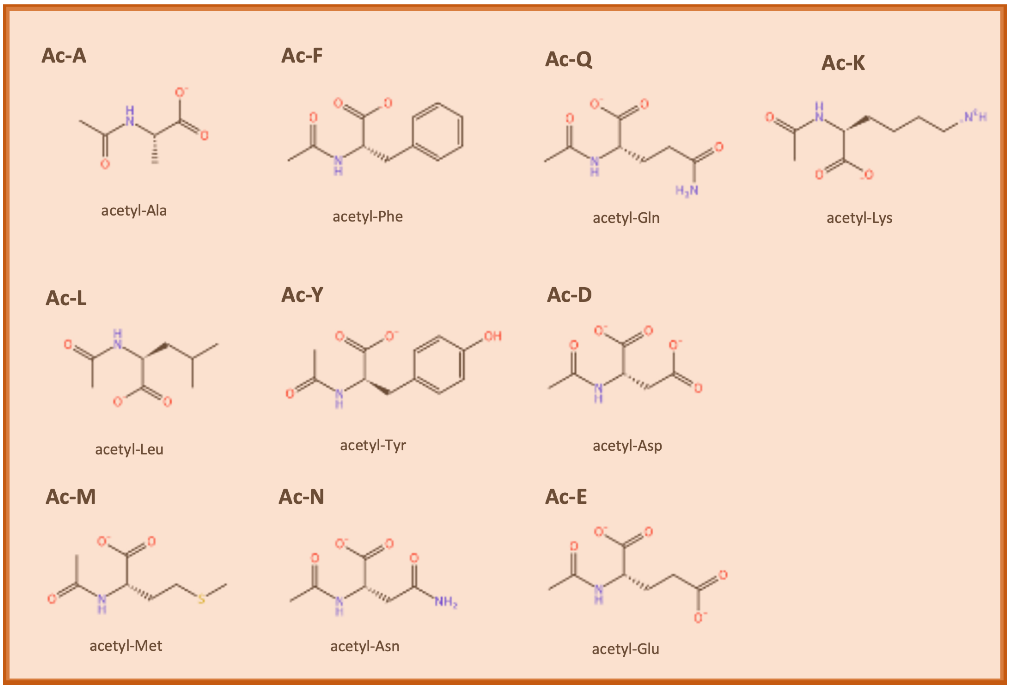

Ac-A
acetyl-Ala
Ac-F
acetyl-Phe
Ac-Q
acetyl-Gln
Ac-K
acetyl-Lys
Ac-L
acetyl-Leu
Ac-Y
acetyl-Tyr
Ac-D
acetyl-Asp
Ac-M
acetyl-Met
Ac-N
acetyl-Asn
Ac-E
acetyl-Glu

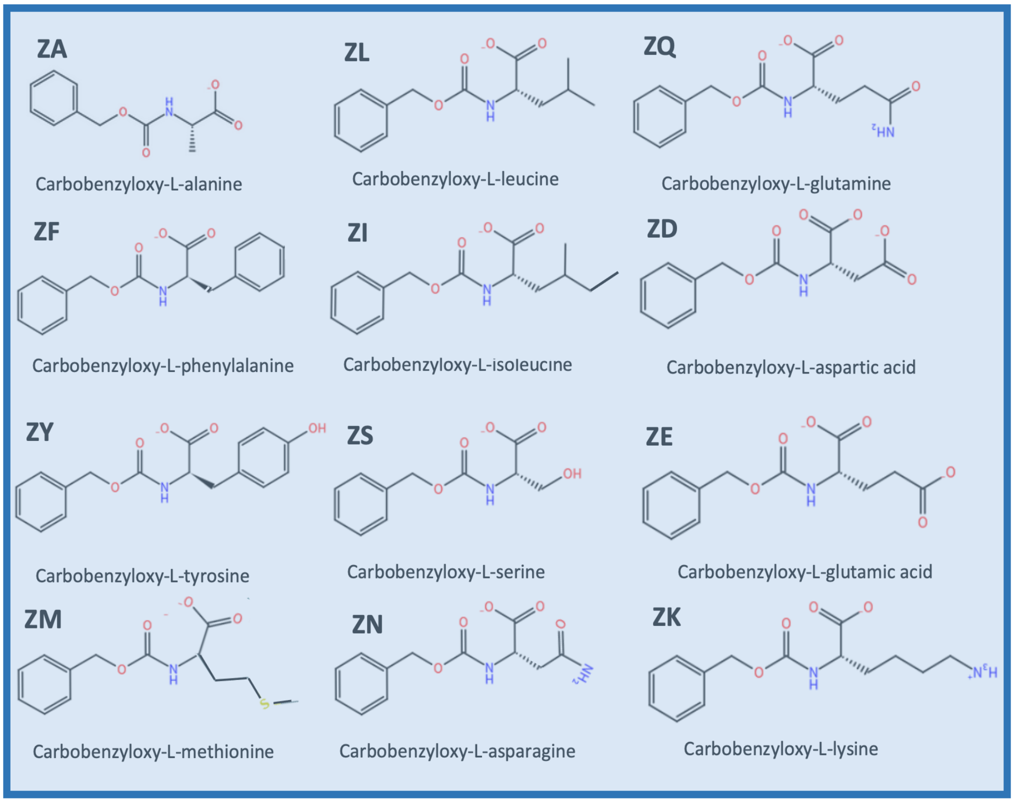

ZA
Carbobenzyloxy-L-alanine
ZL
Carbobenzyloxy-L-leucine
ZQ
Carbobenzyloxy-L-glutamine
ZF
Carbobenzyloxy-L-phenylalanine
ZI
Carbobenzyloxy-L-isoleucine
ZD
Carbobenzyloxy-L-aspartic acid
ZY
Carbobenzyloxy-L-tyrosine
ZS
Carbobenzyloxy-L-serine
ZE
Carbobenzyloxy-L-glutamic acid
ZM
Carbobenzyloxy-L-methionine
ZN
Carbobenzyloxy-L-asparagine
ZK
Carbobenzyloxy-L-lysine

Figure S4 (*cont*)

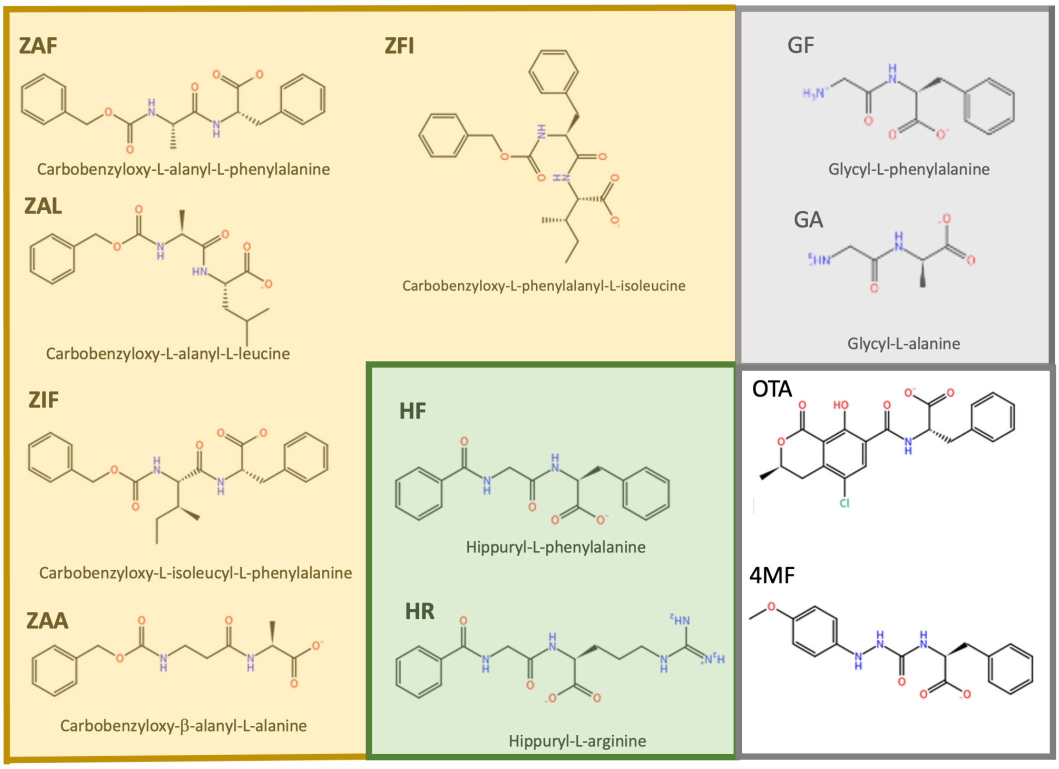

Figure S4. Structures of the substrates used in our study classified into five families: *N*-acetyl-L-amino acid (orange); *N*-acetyl-L-amino acids with one amide bond (blue); *N*-acetyl-L-amino acids with two amide bonds (light brown); hippuryl-L-amino acid (green); L-dipeptides (grey). The name of each substrate appears below its 2D chemical structure. The structures of OTA and 4MF are also shown.

Figure S5

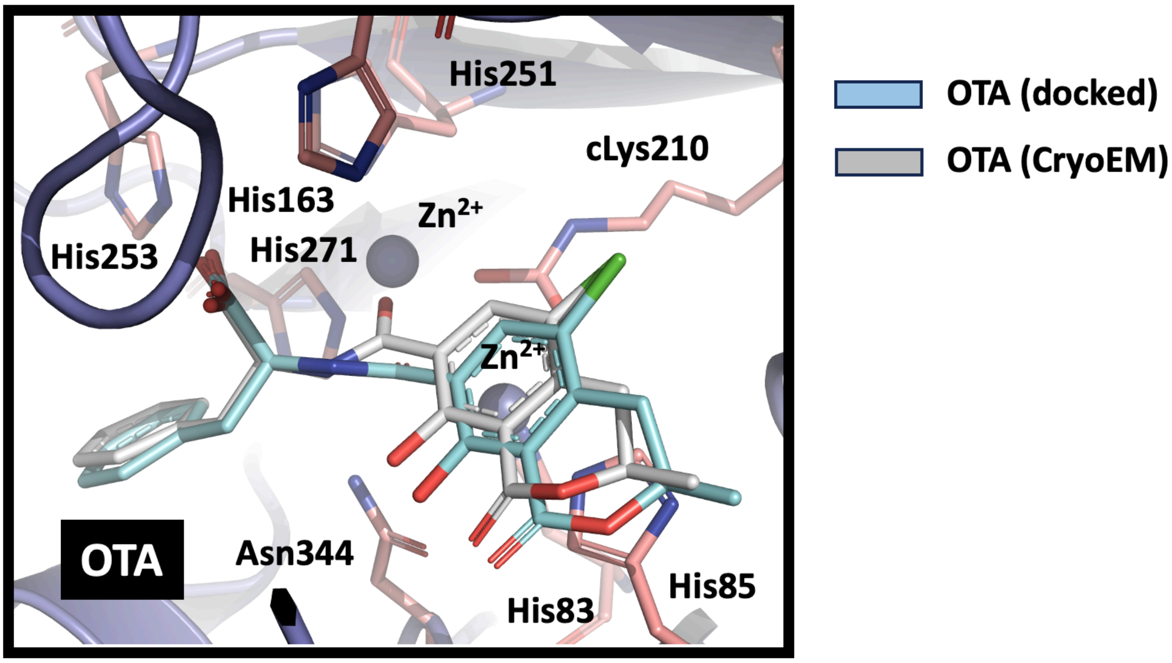

Figure S5. Docking of OTA into the CryoEM 3D structure of the *An*OTA homolog enzyme ADH3 from *Stenotrophomonas acidaminiphila* (*Sa*OTA), and comparison with the experimentally determined structure of the mycotoxin.

Figure S6

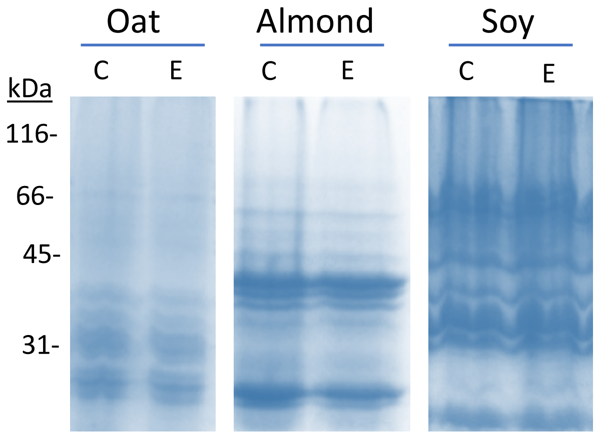

Figure S6. SDS-PAGE analysis of plant-based beverages containing OTA (5 μM) incubated with *An*OTA at 37 ºC for 18 h. Reactions containing *An*OTA are indicated (E). Control reactions without enzyme are also shown (C). The 12.5% gel was stained with Coomassie blue. Molecular mass markers are on the left (SDS-PAGE standards, Bio-Rad).
